# Treatment of asthma: Identification of the practice behavior and the deviation from the guideline recommendations

**DOI:** 10.4103/0970-2113.68315

**Published:** 2010

**Authors:** Parthasarathi Bhattacharyya, Rantu Paul, Saikat Nag, Sujan Bardhan, Indranil Saha, Malabika Ghosh, Ratna Dey, Rana Dey, Sahidul Islam, Dipabali Acharyya (Ghosh)

**Affiliations:** *Institute of Pulmocare and Research, RG Kar Medical College, Kolkata, India*; 1 *Department of Community Medicine, RG Kar Medical College, Kolkata, India*

**Keywords:** Asthma, guidelines, practice behaviour

## Abstract

**Background:**

Despite an exponential development of the understanding of the disease with availability of good therapy and feasibility of good control along with availability of globally accepted guidelines, there remains a significant gap between the guidelines and prevailing practice behavior for treating asthma all over the globe. This perhaps stands as the single most deterrent factor for good asthma care worldwide. The objective of the study is to analyze the asthma prescriptions to find out the available status of the practice behaviour and the deviations from the guideline in asthma practice.

**Materials and Methods:**

The asthma prescriptions of the referred patients presenting to the OPD services of the IPCR, Kolkata were photocopied and collected. They were further analyzed based on the available information upon a format being prepared on four major areas as qualifications, clinical recording habit, practice of evaluating patients, and treatment habit that stands apparent from the prescribed medications. The doctors were divided into three categories as a) MBBS, b) MD/DNB (medicine and respiratory medicine), and c) DM (non respiratory sub-specialities) and statistical analysis has been performed comparing the three groups as per the performance in the four pre-decided areas.

**Results:**

All the groups fall short of any guideline or text of asthma care in all the areas involved.

**Conclusion:**

The practice behaviour of our doctors for asthma care appears deficient in several areas and seems far from guideline recommendations. This needs further evaluation and adoption of appropriate interventions.

## INTRODUCTION

Despite tremendous improvement in knowledge and treatment of asthma and availability of guidelines, the asthma practice behaviour of the physicians concerned is perhaps the most important issue to determine the quality of care for asthma. There is always a gap between the guidelines and practice.[1] Understanding the extent and the factors to make this gap is important in order to bridge it since a poorer health-related quality of life has been observed to be associated in asthma patients with non-guideline treatment in general practice.[2] Hence, understanding the practice behaviour of the physicians treating asthma will be a worthwhile exercise. We collected 100 asthma prescriptions randomly from patients attending our clinic with asthma and analyzed the practice behaviour of the treating doctoral in several areas as clinical recording habit, practice of evaluating a patient, and the treatment behaviour of prescribing different types of medicine as per the available information from the prescriptions. Herein, we present a survey on the asthma prescriptions in the community in order to understand the scenario of guideline adherence by the practicing doctors.

## MATERIALS AND METHODS

It has been a cross sectional and observational study. Patients being referred to our OPD services and diagnosed as asthma on spirometry at the institute were requested to give consent and, thereafter, allowed to make photocopies of the prescriptions being carried by them for the study. These prescriptions were preserved and the available information was charted categorically as a) the qualification of the doctors b) the documentation habit that included the documentation of the diagnosis, vitals (pulse, respiratory rate, blood pressure, arterial oxygen saturation), co-morbidities (if any), and the clinical examination of the respiratory system in whatever form available, c) the investigation habit from the available record that includes advice for spirometry, chest X-ray, X-ray of the para-nasal sinuses, blood sugar, routine hemogram, IgE etc and d) the prescribing habit marked by advice for oral or inhaled medications, oral steroid, and inhaled corticosteroids.

The doctors were divided into three categories as (i) MBBS, (ii) MD or equivalent including MD in respiratory medicine, and (iii) DM in any non respiratory sub-speciality of medicine. The different habits of the three different categories of the doctors were charted systematically and information derived was expressed in percentages. Furthermore, the performance of the three categories was compared statistically with unpaired‘t test’.

## RESULTS AND ANALYSIS

The distribution of doctors in different categories as described above was as follows: (A) MBBS (*n* = 28), (B) post graduates (*n* = 46), and (C) post doctoral (*n* = 26). The comparison between the different categories and the overall situation is tabulated below in Table 1–3.

**Table 1:** Clinical recording habit

	A+B+C (total in %)	A (%)	B (%)	C (%)	*P* value		
					A and B	B and C	A and C
Diagnosis of asthma written on prescription	29.0	8 (28.57)	16 (34.78)	5 (19.23)	0.38	0.13	0.31
Co-morbidity mentioned	14.0	3 (10.71)	8 (17.39)	3 (11.53)	0.32	0.37	0.36
Pulse rate recorded	29.0	6 (21.42)	20 (43.47)	3 (11.53)	0.04	0.006	0.27
Blood pressure recorded	48.0	17 (60.71)	21 (45.65)	10 (38.46)	0.15	0.36	0.08
Saturation recorded	0.0	0 (0.0)	0 (0.0)	0 (0.0)	-	-	-
Respiratory rate recorded	5.0	0 (0.0)	4 (8.69)	1 (3.84)	-	0.38	-
Temperature recorded	4.0	2 (7.14)	1 (2.17)	1 (3.84)	0.32	0.36	0.47
Recording of the findings of chest examinations	36.0	7 (25.0)	20 (43.47)	9 (34.61)	0.08	0.31	0.31

Comparison of the recording habit of the different categories of doctors. The significant *P* values are displayed in bold. (A= MBBS doctors, B= doctors with MD or equivalent qualifications, and C= doctors with DM or equivalent qualifications)

**Table 2:** Practice of evaluating a patient

	A+B+C (total %)	A (%)	B (%)	C (%)	*P* value		
					A and B	B and C	A and C
Routine hemogram	24.0	7 (25.0)	11 (23.91)	6 (23.07)	0.43	0.41	0.43
CxR (PA)	21.0	5 (17.85)	8 (17.39)	8 (30.76)	0.39	0.15	0.21
X-ray of PNS	5.0	2 (7.14)	0 (0.0)	3 (11.53)	-	-	0.46
Sputum AFB test	3.0	2 (7.14)	1 (2.17)	0 (0.0)	0.32	-	-
Sugar PP/ F	4.0	1 (3.57)	3 (6.52)	0 (0.0)	0.49	-	-
ECG	5.0	0 (0.0)	3 (6.52)	2 (7.69)	-	0.38	-
Spirometry/ PFT	9.0	1 (3.57)	5 (10.86)	3 (11.53)	0.24	0.38	0.27

Comparison of the evaluation habit of the different categories of doctors. The significant *P* values are displayed in bold. (A= MBBS doctors, B= doctors with MD or equivalent qualifications, and C= doctors with DM or equivalent qualifications)

**Table 3:** Prescription habit

	A+B+C (total %)	A (%)	B (%)	C (%)	*P* value		
					A and B	B and C	A and C
Inhaler only	31.0	5 (17.85)	20 (43.47)	6 (23.07)	0.02	0.07	0.44
SABA alone*	7.0	1 (3.57)	5 (10.86)	1 (3.84)	0.24	0.27	0.25
ICS+SABA*	7.0	1 (3.57)	3 (6.52)	3 (11.53)	0.49	0.38	0.27
ICS+LABA*	17.0	3 (10.71)	12 (26.08)	2 (7.69)	0.09	0.06	0.46
Oral only	19.0	6 (21.42)	5 (10.86)	8 (30.76)	0.18	0.04	0.31
Combination of inhaler and oral	27.0	6 (21.42)	14 (30.43)	7 (26.92)	0.28	0.48	0.43
No medication	24.0	12 (42.85)	7 (15.21)	5 (19.23)	0.009	0.45	0.06

* Percentage among inhaler users, Comparison of the prescription habit of the different categories of doctors. The significant *P* values are displayed in bold. (A= MBBS doctors, B= doctors with MD or equivalent qualifications, and C= doctors with DM or equivalent qualifications)

While analyzing the data, we notice a striking deficit in performance from the recommended practice in all the categories of doctors in all the areas of observations. There is a statistically significant difference in the practice behaviour for recording of pulse rate in favor of post graduate doctors (category B) compared to the other categories (A and C). However, the latter group (category C, the non-pulmonary DM doctors) has practiced advising chest X-ray (PA) and prescribed oral medication (p<0.03) in a significantly higher proportion. Although not significant, blood pressure measurement is apparently done more frequently by MBBS doctors compared to the other two categories. The evaluation habit of the doctors also falls short of the expected; overall the routine hemogram and chest X-ray were advised by about one fourth (24 %) and one fifth (21 %) of the prescribers. The post doctoral doctors were most smart to ask a chest X-ray and the overall consideration of allergic rhinitis appears low from the fact that only 5 % doctors asked a X-ray of the para nasal sinus. The prescription of the use of inhales (alone or in combination with oral) has scored out higher than advice for oral medication alone (58% versus 19%). When looked for, the use of inhaled medication alone, combination products (ICS+LABA) outscore the use of ICS+ SABA (44% versus 6 %); inhalers are prescribed more by the post graduate doctors.

## DISCUSSION

The overall impression from the available data is that there is still a huge dearth between the published guideline for asthma therapy and the expressed practice behaviour of doctors in this part of the developing world. The deficit is known as universal and seemingly unrelated to the level of qualification in our study. Thus, the behaviour noticed may not necessarily indicate the lack of knowledge regarding asthma but certainly points to the failure of achievement of guideline provided standard. It is not possible to implicate the reason which could be largely external and circumstantial for the doctors to observe a better documentation, evaluation, and prescription habits but, nevertheless, one cannot exclude a certain degree of lacunae in understanding the disease. The exceedingly low use of peak flow measurement or /and spirometry (overall 9.0 %), failure to suspect asthma in about one fourth (24 %) of patients (were kept off any anti asthma medication) probably points to low level of understanding or motivation among the doctors. On the contrary, the level of the use of inhalational products either alone or in combination with oral medications was relatively impressive (58 %), along with the use of inhaled corticosteroid in combination with LABA; this suggests that the fundamental basis of use of anti-inflammatory medications in asthma has been already incorporated in the practice behaviour of the physicians concerned. If this is taken as a marker of guideline acquaintance, we may need to put more stress on the circumstantial and patient-related factors for such deficient practice behaviour.

Several recommended asthma guidelines are available to help the physicians to treat the disease better on evidence-based information. However, the ability of guidelines to change a physician’s behavior or the patient outcomes has been limited.[3] For asthma too, it is common to find a mismatch between the guideline opinion and the course of action by a general practitioner.[4] The fault of non adherence to a guideline involves the treating doctors and the patients as well and it expands irrespective of the patients’ socioeconomic status.[5] In a survey among pediatricians, 171 comments about barriers to adherence to a guideline have been noted. These perceived barriers could be related to a) the physician concerned (as the age of the physician, lack of awareness / familiarity and / or agreement, lack of self-efficacy, lack of outcome expectancy, and inertia of previous practice etc., b) the external barriers owing to environmental influence which could be cultural or socioeconomic factors, and c) several patient-related issues.[6] Some barriers are identified as lack of familiarities[7] and lack of agreement[8] while economic disincentives to perform recommendations, patient noncompliance, and inadequate time or resources to perform recommendations are hypothesized to be the other important barriers.[9,10]

The prescribing habits of different categories of drugs are different at different places. A study to explore and compare treatment decisions and the influence of specific patient characteristics on the management of asthma in five different European countries revealed that there is a significant difference in the prescribing habit of oral or inhaled corticosteroid and antibiotics.[11] Physicians are found to take account of perceived rather than evidence-based notions in choosing a drug from a list of alternatives; issues such as efficacy, personal experience, expected adverse drug effects, user-friendliness and cost aspects in mind are seen to influence the prescriptions.[12] Although the patient characteristics are proposed to be of primary relevance when understanding clinical decision making in general,[13] situational factors that vary in different health care settings can influence treatment decisions.[14] Ironically, it has been noted that the professional organizations have invested much heavily in the development of practice guidelines compared to their involvement in understanding what is required to apply these guidelines in practice.[15]

This small study has a lot of limitations; several factors as the number of the prescriptions studied (was not large), the disease control status and the lung function at the presentation, the reason for presenting to us (being referred or not), the educational and socioeconomic status of the patients, the practicing area of the doctors (urban/rural) etc. were not taken into consideration. The dose issue of the inhaled medication (ICS or non-ICS) is also not incorporated. It is not possible to ensure that the available record is the true one since some of the physicians may have been keeping their personal record separately and have written only the medications in the prescription. Moreover, the exact status of the patients presenting at the doctors is not known although the reason of attending our OPD was presumably a non satisfactory response to therapy for most of the patients. It is also possible that, in some occasions, the referring physician had just given a set of advice before referring the patient to us. Therefore, the prescriptions scanned may not be the exact representation of the actual status of the prescribing habit in our community and have some deviations from the exact prescription habit of our doctors.

In our observation, the available information provides limited insight into the causes of non adherence but it amply elaborates the reality in asthma care in this part of the world and impresses upon the need of well planned and elaborate effort to look further into the dimension and factors for non adherence.

## CONCLUSIONS

Our experience from this small data suggests that there is huge gap between the guideline and the practice behaviour of our physicians. Further studies are needed to assess in-depth the factors responsible for the inappropriate and inadequate practice behaviour of our doctors to find out ways to eliminate the deficiencies to help millions of our patients.
